# Risk stratification for postoperative complications after CRS and HIPEC in recurrent ovarian cancer patients: a comparative analysis of logistic regression and machine learning models

**DOI:** 10.1038/s41598-025-33049-9

**Published:** 2025-12-18

**Authors:** Jakub Litwiński, Katarzyna Gęca, Izabela Świetlicka, Łukasz Wiśniowski, Wojciech Polkowski, Magdalena Skórzewska

**Affiliations:** 1 Department of Surgical Oncology, Medical University of Lublin, Lublin, Poland; 2 Department of Biophysics of Biological Structures and Systems, University of Life Sciences in Lublin, Lublin, Poland; 3 Department of Cardiology, Medical University of Lublin, Lublin, Poland

**Keywords:** Oncology, Risk factors

## Abstract

Cytoreductive surgery (CRS) combined with hyperthermic intraperitoneal chemotherapy (HIPEC) is associated with improved survival in recurrent ovarian cancer (ROC) but carries a high risk of postoperative complications. Accurate perioperative risk stratification remains an unmet need. To develop and internally validate a perioperative risk model for postoperative complications in ROC patients using information available by the end of surgery (pre- and intra-operative data), and to compare logistic regression (LR) and artificial neural networks (ANN) as possible predictive models. A retrospective analysis of 71 patients treated with CRS and HIPEC between 2011 and 2022 was performed. Clinical, surgical, and perioperative variables were analysed. Predictors were restricted to variables available at or before the end of the index operation, which prevents information leakage by using only data available at prediction time. LR and ANN models were developed and assessed with cross-validation. Performance reporting followed TRIPOD (Type b) and TRIPOD + AI, with Brier score, and calibration slope/intercept from out‑of‑fold (OOF) predictions. Thresholded metrics (accuracy, precision, recall, F1 score, and the area under the receiver operating characteristic curve, AUC) were summarised at a prespecified probability cut-off. Exploratory univariate odds ratio analyses with Holm-adjusted p-values were used to explore procedure–complication associations. Postoperative complications occurred in 45% of patients. LR identified blood loss (*p* = 0.005) and number of procedures (*p* = 0.042) as significant predictors of complications. The LR model achieved an accuracy of 66.2%, precision of 64.3%, recall of 56.2%, F1 score of 60.0%, and AUC of 0.700. The ANN model achieved an accuracy of 97.2%, precision of 94.3%, recall of 100%, F1 score of 97.1%, and AUC of 0.967. Hysterectomy with adnexa (OR = 11.67, *p* = 0.035) and metastasectomy (OR = 7.42, *p* = 0.042) were significantly associated with higher postoperative complication rates. ANN demonstrated superior predictive performance compared to LR in identifying postoperative complications after CRS and HIPEC, as indicated by ROC analysis. Combining traditional statistical modelling with modern machine learning may enhance ROC for perioperative risk stratification after CRS with HIPEC. A well-calibrated, interpretable LR model together with a highly discriminative ANN could enable more tailored allocation of intensive care resources and earlier identification of high-risk patients, potentially improving the safety of this demanding but beneficial treatment. However, external multicentre validation is required before clinical implementation.

## Introduction

Ovarian cancer (OC) remains one of the leading causes of gynaecological cancer mortality worldwide, with approximately 313,000 new cases and 207,000 deaths reported annually^1^. Despite advances in surgical techniques and systemic therapies, the 5-year survival rate for patients with advanced-stage disease remains poor, often below 40%^2^. Late-stage diagnosis and intrinsic tumour biology contribute to the dismal outcomes. The recurrence rate in epithelial OC remains high, with up to 70–80% of patients with advanced-stage disease experiencing relapse despite initial response to therapy.

Cytoreductive surgery (CRS) combined with hyperthermic intraperitoneal chemotherapy (HIPEC) has emerged as a promising strategy for selected patients with advanced and recurrent ovarian cancer (ROC)^3^. Several studies suggest that maximal CRS followed by HIPEC improves progression-free (PFS) and overall survival (OS) compared to systemic therapy alone^3–5^. However, the benefits of HIPEC remain a subject of ongoing debate, particularly regarding its role in primary versus recurrent disease and its impact on morbidity^5^.

CRS combined with HIPEC is associated with a substantial risk of serious postoperative complications, including infectious, haematological, gastrointestinal, and respiratory events^6^. These complications can delay or prevent the initiation of adjuvant chemotherapy, thereby negatively influencing overall prognosis. Therefore, improving patient selection through accurate risk stratification is a critical unmet need.

Traditional predictors of postoperative complications, such as the Peritoneal Carcinomatosis Index (PCI), completeness of cytoreduction (CCR), and American Society of Anesthesiologists Physical Status Classification System (ASA) performance status, offer some guidance but lack sufficient precision^7^. Machine learning (ML) approaches, particularly artificial neural networks (ANNs), offer the potential to capture complex, non-linear interactions among clinical variables that traditional statistical models may overlook.

The objective of this study was to develop and internally validate predictive models for postoperative complications in patients with recurrent ovarian cancer (ROC) undergoing cytoreductive surgery (CRS) combined with hyperthermic intraperitoneal chemotherapy (HIPEC). The models were designed to predict the risk of complications at the end of surgery, before the postoperative disposition decision, using pre- and intra-operative data. We hypothesised that an artificial neural network (ANN)-based model would outperform logistic regression (LR) in predicting postoperative complications. Improved perioperative risk prediction could support real-time decision-making and enable more individualised postoperative management.

## Materials and methods

This retrospective observational study was conducted after approval from the Institutional Review Board (Bioethical Committee of the Medical University of Lublin, Ethics Code: KE—0254/331/2018). Between April 2011 and November 2022, 300 HIPEC procedures were performed at the Department of Surgical Oncology, Medical University of Lublin. Of these, 71 HIPEC procedures (23.7%) were carried out in patients with ROC, whose data were included in the present analysis.

### Inclusion criteria

- Age ≥18 years.
- Histologically confirmed diagnosis of recurrent epithelial OC.
- Status post-surgery for primary OC with adjuvant platinum- based CTH.
- Recurrence confirmed via radiology with/without elevated CA-125 and HE4 levels.
- Al least one measurable target lesion per RECIST v 1.1.
- ECOG Performance Score of 0–2, indicating ability for self-care and ambulatory for over 50% of waking hours.
- Adequate organ and bone marrow function for CRS and HIPEC treatment.
- Deemed suitable for CRS and HIPEC by a multidisciplinary team.
- Written informed consent for treatment.

### Exclusion criteria

- Diagnosis of non-epithelial OC like germ cell or stromal tumours.
- Stage IV disease marked by extra-abdominal metastasis.
- Previous treatment with CRS and HIPEC for OC.
- Serious cardiac, lung, kidney, or liver conditions posing surgery risks.
- Other active primary cancers.
- ECOG status of 3 or higher, indicating severe incapacitation.
- Underlying medical or psychiatric conditions that will make the administration of therapy hazardous.
- Dementia, psychiatric, or substance abuse disorders that would interfere with treatment.
- Known untreated, symptomatic, or actively progressing central nervous system metastases.
- Known acute hepatitis B, known chronic hepatitis B infection with active untreated disease, or known hepatitis C infection.
- Life expectancy under 6 months.

### Platinum sensitivity

Platinum-sensitive disease is defined as a recurrence occurring more than 6 months after completion of standard first-line chemotherapy^8^. Patients with platinum-sensitive recurrence were treated with platinum-based chemotherapy. In cases of platinum-resistant recurrence, treatments included paclitaxel, topotecan, gemcitabine, etoposide, or liposomal doxorubicin.

### The Peritoneal Carcinomatosis Index and cytoreduction completeness

The PCI was used to stage the peritoneal advancement of disease^9^. This index assigns a score ranging from 0 to 39 points, representing the size of tumour lesions across 13 regions of the peritoneal cavity. The CCR was evaluated on a four-point scale: CC0 represents complete cytoreduction, CC1 indicates residual tumour nodules smaller than 2.5 mm, CC2 refers to residual nodules between 2.5 mm and 2.5 cm, and CC3 signifies residual lesions larger than 2.5 cm. The effective use of HIPEC depends on achieving either CC0 or CC1 resections, as optimal cytotoxicity can only be expected when there is no visible tumour (CC0) or minimal residual nodules (no larger than 2.5 mm). CRS, including peritonectomy, is performed in every anatomical region of the abdomen and/or pelvis where peritoneal metastases (PM) are present. The extent of the resection varied significantly depending on the quantity and location of PM.

### Surgical and HIPEC procedures

PCI and CCR scores were assessed intraoperatively by the surgical team. CRS aimed for complete (CC-0) or near-complete (CC-1) resection. HIPEC was administered intraoperatively using either the open “Coliseum” or closed technique^10^. HIPEC was administered with a perfusion rate ranging between 4 and 11 L per minute while maintaining a target intraperitoneal temperature of 42–43 °C, facilitated by the SunChip^®^ 1.0 system (Gamidatech, Eaubonne, France). Two cytotoxic agents were employed for intraperitoneal perfusion: Mitomycin C at a dose of 30 mg, dissolved in 0.9% NaCl solution for a duration of 60 min, or Oxaliplatin at a dose of 300 mg/m², dissolved in 5% glucose solution, administered over a period of 30–45 min. The choice of cytostatic agent was made during MDT evaluation, based on platinum sensitivity status and clinical factors.

### Charlson comorbidity index

The Charlson Comorbidity Index (CCI) is a widely used scoring system that estimates a patient’s one-year mortality risk based on the presence and severity of comorbid conditions. Each condition is assigned a weight, and the total score correlates with the risk of mortality. Conditions such as heart disease, cancer, liver disease, and diabetes are included in the CCI, with higher scores indicating a greater risk of death^11^. The CCI is commonly used in clinical research and patient care to adjust for comorbidities when evaluating outcomes.

### Prediction timepoint, outcome definition, and prespecified assumptions

The primary outcome was any postoperative complication occurring during the index CRS + HIPEC admission up to hospital discharge, graded according to the Clavien–Dindo classification. For each patient, the highest recorded Clavien–Dindo grade before discharge defined the binary outcome (complication: yes/no). Clavien–Dindo grade I events were retained because even minor deviations can delay discharge and increase resource use after the studied procedure. Complications were ascertained from the inpatient electronic health record (ICU and ward progress notes, anaesthesia and operative records, medication orders, and the discharge summary). The prediction time point was defined at the end of surgery, before the postoperative disposition decision. To avoid incorporation bias and policy dependence, immediate postoperative disposition and ICU length of stay were not used as predictors. No in‑hospital deaths occurred during the observation window. Missingness was audited across all variables, and none were observed. Follow‑up during the index admission was complete through discharge.

### Model construction

Prediction was defined at the end of surgery, before the postoperative disposition decision, using only pre- and intra-operative variables. The data included age, CRS time (per 10 min), CCR, cytostatic type, method, HIPEC time (per 10 min), number of procedures, ASA, blood loss (per 100 mL), and blood transfusion. To respect events‑per‑parameter constraints and avoid collinearity with surgical complexity, we summarised procedures by the ‘number of procedures’ variable and did not include individual procedure indicators in the predictive models. The relationship between the variables describing the features of the surgery and the probability of complications was analysed using logistic regression (LR) and artificial neural networks (ANNs). Given the small, single‑centre cohort (71 patients, 32 events), LR degrees of freedom were constrained a priori, and a stratified 5‑fold cross‑validation was used for internal validation, consistent with TRIPOD type 2b reporting. Accordingly, the prespecified LR included age, CRS time, surgical method (reference: closed), HIPEC time, number of procedures, ASA (3 vs 2), and blood loss (per 100 mL). Correlated candidates, namely CCR, cytostatic agent, and transfusion, were excluded to avoid redundancy with surgical complexity and blood loss, which are already represented in the model. For comparability, the ANN was trained on the full prespecified candidate set (i.e., including CCR, cytostatic agent, and blood transfused), with continuous predictors standardised (z-score) within each training fold, while categorical variables were one-hot encoded. Given the small cohort, the network architecture was kept compact, all hyperparameters were prespecified and held fixed, and no additional hyperparameter tuning was undertaken. The MLP network was trained with the Broyden-Fletcher-Goldfarb-Shanno (BFGS) algorithm^12^ for up to 1000 epochs using sum-of-squares error, with L2 weight decay. Internal validation followed the same stratified 5-fold scheme as for LR, and all preprocessing and Platt (logistic) recalibration were performed on the training split and applied to the held-out fold. Classification was summarised at a prespecified threshold of 0.50 (no high/low acceptance–rejection rules).

### Statistical analysis

The collected data were analysed concerning their completeness and nature. No missing values were observed (0%) in any of the analysed variables. Continuous variables were presented as means with 95% confidence intervals or as medians with the 25th and 75th percentiles (Q1-Q3). Categorical variables were summarised as counts and percentage shares (%).

LR and ANNs were used to assess the probability of postoperative complications. For LR, a prespecified, parsimonious perioperative predictor set was used to constrain degrees of freedom, given the small event count. Predictors were chosen a priori on clinical grounds (age, CRS time, surgical method, HIPEC time, number of procedures, ASA status, and blood loss). The LR model related the log odds (logit) of the event probability to a linear combination of predictors, with parameters estimated by maximum likelihood. For the ANN, the same perioperative predictor set was used. Continuous inputs were standardised (z-scores) before fitting and used as input to the tested algorithms.

Internal validation used stratified 5-fold cross-validation with a fixed seed, applying the same folds to both models. All preprocessing was performed within each training fold and then applied to the corresponding validation fold to avoid information leakage. Out-of-fold (OOF) predictions were concatenated across folds and used for all performance summaries.

Models’ discrimination abilities were assessed using a confusion matrix and related statistics (accuracy, precision, recall and F1 Score) as well as the receiver operating characteristic (ROC) curve and the area under the ROC curve (AUC-ROC). Threshold‑based classification metrics were computed from OOF predictions at a prespecified probability cut‑off of 0.50. Overall probabilistic accuracy was measured using the Brier score. Calibration was examined numerically via calibration‑in‑the‑large (intercept) and calibration slope (from logistic recalibration of OOF probabilities) and the Integrated Calibration Index (ICI/E_avg_), and visually using an OOF calibration plot with the logistic recalibration line and a non‑parametric smooth of observed vs. predicted risk (LOESS). Where relevant, 95% confidence intervals for OOF metrics were obtained by bootstrap resampling of OOF predictions (B = 2000). For the ANN, logistic (Platt) scaling was fitted within each training fold and applied to the corresponding validation fold. Additionally, kernel density plots of out-of-fold predicted probabilities were used to analyse the distribution of predicted risk of complications and to assess central tendency and variability. The Hosmer–Lemeshow statistic was reported descriptively only, whereas Tjur’s R² showed the difference in mean predicted probabilities between outcome groups, providing an intuitive measure of discrimination.

As an exploratory, hypothesis‑generating analysis, we assessed the association between individual surgical procedures and complications using univariate odds ratios from 2 × 2 tables (exposure: procedure performed yes/no; outcome: complication yes/no). Pearson’s χ² (or Fisher’s exact when expected counts < 5) provided p‑values. To account for multiple comparisons across procedures, p‑values were Holm‑adjusted (with FDR reported in sensitivity analysis). These analyses were not used for model training.

All simulations were conducted in R (4.4.2) using RStudio (2024.12.1 Build 563, Posit Software, PBC, Boston, MA; URL: http://www.rstudio.com/) with key packages pROC (1.18.5), rms (8.0–0, calibration and ICI), caret (7.0–1, resampling/preprocessing), boot (1.3–31, bootstrap CIs), ggplot2 (3.5.1, figures), and base stats (glm/loess), STATISTICA 13.0 (TIBCO Software, Palo Alto, CA, USA), and OriginPro 2022 (OriginLab, MA, USA). Reporting follows TRIPOD Type 2b recommendations (model development with internal validation via stratified 5‑fold cross‑validation), including explicit handling of missingness, internal validation strategy, and discrimination and calibration summaries.

### Data characteristic

A total of 71 female patients with ROC underwent CRS combined with HIPEC. The mean age of the cohort was 54.1 years (95% CI: 52.09;56.18). The median estimated intraoperative blood loss was 100 mL (Q1-Q3: 0–200). CRS procedures lasted, on average, 159.9 min (95% CI: 146.63;173.09), while HIPEC perfusion lasted 39.3 min on average (95% CI: 36.40;42.20).

Postoperatively, the median length of hospital stay was 7 days (Q1-Q3: 6–9). The median CCI score was 8.00 (Q1-Q3: 0.00;20.90), indicating a moderate comorbidity burden in the study population (Table 1).

**Table 1:** Continuous variable characteristic.

Characteristics	Mean (95% CI)	Median (Q1;Q3)
Age (years)	54.14 (52.09;56.18)	-
CRS time (minutes)	159.86 (146.63;173.09)	-
HIPEC time (minutes)	39.30 (36.40;42.20)	-
Blood loss (mL)	-	100.0 (0;200.0)
Blood transfused (units)		0.50 (0.00;1.00)
Hospital stay (days)	-	7.00 (6.00;9.00)
CCI	-	8.00 (0.00;20.90)

Continuous variables are summarised as mean (with 95% confidence intervals, CI) when approximately symmetric and as median (Q1–Q3) when skewed; Q1 and Q3 denote the 25th and 75th percentiles.

Two cytostatic agents were used during HIPEC: Mitomycin C in 42% of cases and Oxaliplatin in 58%. The open technique was employed in 54% of procedures, and the closed method in 46%. Optimal cytoreduction (CCR 0) was achieved in 80% of cases; CCR 1 in 15.5%, CCR 2 in two patients, and CCR 3 in one patient. The majority of patients (77%) were classified as ASA grade 3, with the remaining (23%) as ASA grade 2.

Each patient underwent between 1 and 7 surgical procedures during CRS. The most frequently performed interventions included pelvic peritonectomy (65%), liver capsule resection (52%), and parietal peritonectomy (40%). Hysterectomy with adnexa was performed in 16% of patients. This decision was primarily based on oncological considerations, including suspected residual or recurrent disease in the uterus or adnexa, incomplete prior cytoreduction, or to facilitate comprehensive peritoneal access during cytoreduction.

Postoperative complications occurred in 32/71 (45.1%) patients during the index admission up to discharge. By worst Clavien–Dindo grade, 21/71 (29.6%) were grade I–II and 11/71 (15.5%) were grade III–V. ICU admission occurred in 6/71 during the index stay (Table 2). No in‑hospital deaths occurred.

**Table 2:** Categorical variables counts and percentages.

Characteristic		Number of patients *n*	Percentage
Cytostatic	Mitomycin C	30	42
	Oxaliplatin	41	58
Surgery method	Open	38	54
	Closed	33	46
CCR	0	57	80
	1	11	15
	2	2	3
	3	1	2
ASA	2	16	23
	3	55	77
Pelvic peritonectomy	Yes	46	65
	No	25	35
Diaphragmatic peritonectomy	Yes	18	25
	No	53	75
Parietal peritonectomy	Yes	28	40
	No	43	60
Liver capsule resection	Yes	37	52
	No	34	48
Greater omentum resection	Yes	27	38
	No	44	62
Lesser omentum resection	Yes	5	7
	No	66	93
Hysterectomy with adnexa	Yes	11	16
	No	60	84
Appendectomy	Yes	13	18
	No	58	82
Metastasectomy	Yes	13	18
	No	58	82
Resection of the rectum/sigmoid colon	Yes	9	13
	No	62	87
Splenectomy	Yes	5	7
	No	65	93
Right hemicolectomy	Yes	1	2
	No	70	98
Cholecystectomy	Yes	6	8
	No	65	92
Small intestine resection	Yes	8	11
	No	63	89
Pancreas resection	Yes	1	2
	No	70	98
Excision of cancer nodules	Yes	64	90
	No	7	10
Blood loss requiring transfusion	Yes	12	17
	No	59	83
ICU admission	Yes	6	8
	No	65	92
Complications	Yes	32	45
	No	39	55
Clavien scale	I-II	21	30
	III-IV	11	15
	No complications	39	55

Values are n (percent) of all patients (*n* = 71). ICU admission refers to any postoperative admission to the intensive care unit during the index admission, from admission through discharge. Complications are classified by the worst Clavien–Dindo grade recorded before discharge. ASA — American Society of Anesthesiologists physical status; CCR — completeness of cytoreduction; ICU — intensive care unit.

### Models description and comparison

The logistic regression model (Table 3) showed significant contributions (*p* < 0.05) from two variables: the blood loss (per 100 mL) and the number of procedures, while ASA showed marginal significance (*p* = 0.073). Odds ratios (ORs) indicate that higher blood loss and a greater number of procedures are associated with a 63.2% and 43.1% increase in the odds of possible complications, respectively. Higher ASA was associated with an increased risk of complications, although this effect was only marginally significant and characterised by high uncertainty (wide CI). The remaining covariates did not substantially impact the likelihood of complications. The overall model was statistically significant (likelihood-ratio χ² = 25.63, df = 7, *p* < 0.001), indicating that the perioperative predictors collectively improved model fit over the intercept-only model. The model showed acceptable calibration (Hosmer-Lemeshow = 9.223, *p* = 0.324). The coefficient of determination of 32% (according to Tjur’s R Square) indicates a moderate ability to discriminate between the two outcome categories, as the average predicted probability for positive cases is 32% points higher than for negative cases.

**Table 3:** Results of logistic regression analysis for individual independent variables.

Parameter estimates	Variable	Estimate (95% CI)	*p*-value	OR (95% CI)
β_0_	Intercept	-5.850 (-6.88;-0.99)	-	-
β_1_	Age (years)	0.017 (-0.057;0.096)	0.667	1.021 (0.944;1.067)
β_2_	CRS time (per 10 min)	-0.059 (-0.181; 0.053)	0.311	0.942 (0.834;1.045)
β_3_	Method (ref. closed)	0.084 (-0.360;0.823)	0.894	1.092 (0.314;2.780)
β_4_	HIPEC time (per 10 min)	-0.004 (-0.050;0.045)	0.879	0.996 (0.948;1.055)
β_5_	Number of procedures^**^	0.355 (0.127;0.777)	0.041	1.431 (1.009;1.981)
β_6_	ASA^*^ (ref. 2)	1.341 (0.391;2.379)	0.073	3.826 (0.997;6.201)
β_7_	Blood loss^**^ (per 100 mL)	0.488 (0.195;0.897)	0.005	1.632 (1.220;2.450)

*** - significance for *p* < 0.001, ** - significance for *p* < 0.05, * - significance for *p* < 0.1, CI – confidence intervals, SE – standard error, OR – odds ratios.

The multilayer perceptron (MLP), chosen to classify cases into one of two groups, was built with one hidden layer (3 units, logistic activation) and a logistic output for binary classification. Out-of-fold validation accuracy averaged 97.2% across the five stratified folds, confirming adequate learning without severe overfitting. Using the same peri‑ and intra‑operative predictors, the ANN produced more polarised risk estimates than LR, with fewer borderline probabilities and more cases assigned near 0 or 1. Full numerical summaries of discrimination, calibration, and threshold‑based classification are reported in Table 4; Figs. 2 and 3.

Both models underwent the same internal validation procedure and were evaluated with pooled out-of-fold predictions. AUC, Brier score, calibration slope/intercept, and thresholded metrics (accuracy, sensitivity, specificity, PPV, NPV) at a prespecified cut-off were reported. All estimates are derived from validation, not from the training data.

**Fig. 1:**
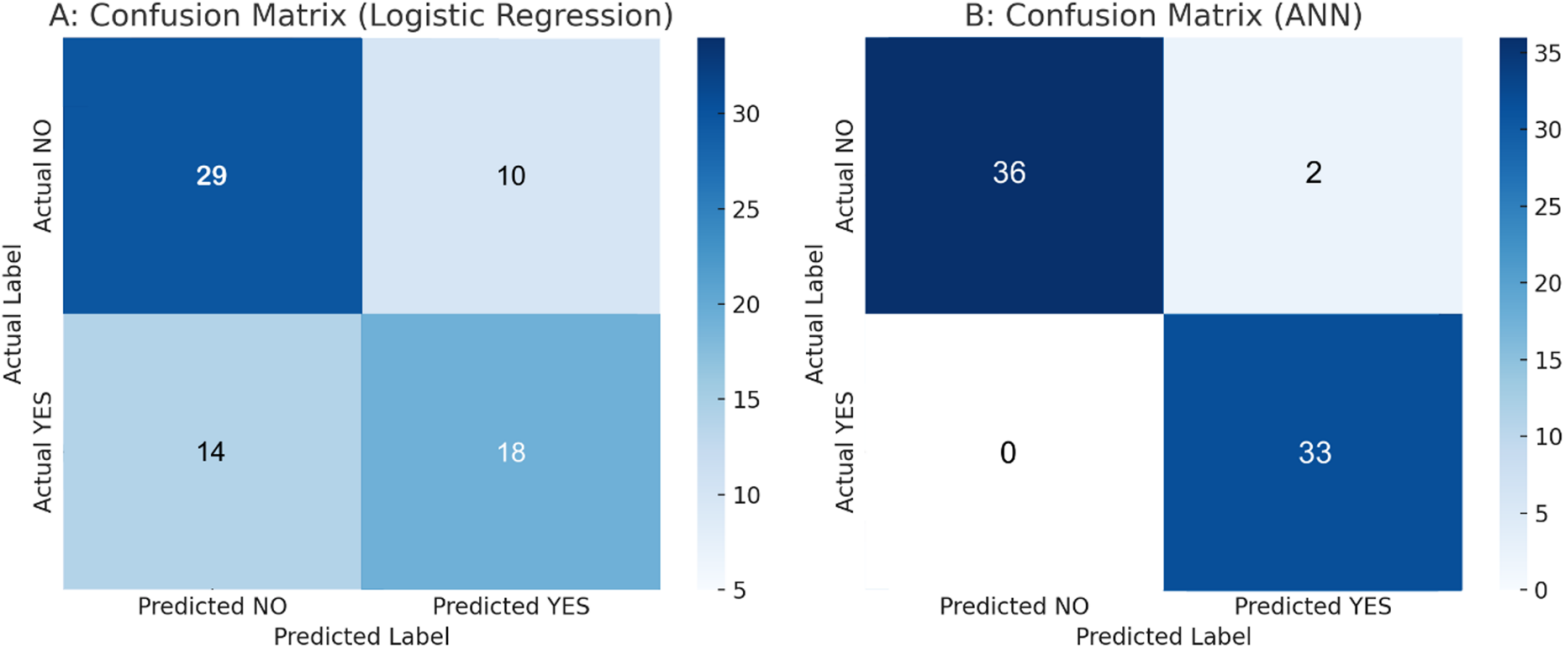
Confusion matrix heatmaps for LR (**A**) and for ANN (**B**) from OOF predictions at threshold 0.50.

The LR model misclassified 24 observations, including 14 positives as negatives and 10 negatives as positives (Fig. 1). The proportion of correctly classified cases (accuracy) using the constructed model (Table 4) was 0.662, with precision (the proportion of correct positive predictions) 0.643, recall (the proportion of actual positives that are correctly identified) 0.562, F1 Score 0.600, and NPV 0.674.

The ANN classifier achieved 0.972 accuracy, a positive predictive value (precision) of 0.943, correctly classifying all positive cases, a proportion of actual positives correctly identified (recall, sensitivity) of 1.00, and an F1 Score of 0.971. The above results show that the model has a low false-positive rate, is perfect at detecting true positives, and maintains a solid balance between precision and recall, indicating that it achieves both a low false-positive rate and a perfect true-positive detection rate.

**Table 4:** Out‑of‑fold validation performance of logistic regression (LR) and artificial neural network (ANN): discrimination, calibration, and classification metrics (threshold = 0.50).

Metrics	LR (95% CI)	ANN (95% CI)
AUC	0.700 (0.565;0.823)	0.967 (0.967;1.000)
Brier score	0.189 (0.149;0.264)	0.235 (0.169;0.297)
Intercept	-0.052 (-0.121;0.046)	-0.135 (-0.267;0.361)
Slope	0.972 (0.821;0.998)	0.857 (0.718;0.987)
ICI (E_avg_)	0.084 (0.067;0.124)	0.124 (0.072;0.222)
Accuracy	0.662 (0.549;0.775)	0.972 (0.943;0.998)
Precision (PPV)	0.643 (0.474;0.818)	0.943 (0.889;0.987)
Sensitivity (recall, TPR)	0.562 (0.387;0.724)	1.000 (0.963;1.000)
Specificity (TNR)	0.744 (0.600;0.878)	0.947 (0.873;0.976)
F1-score	0.600 (0.440;0.737)	0.971 (0.941;1.000)
NPV	0.674 (0.533;0.805)	1.000 (0.981;1.000)

ICI – Integrated Calibration Index, PPV – positive predictive value, TPR – true positive rate, TNR – true negative rate, NPV – negative predictive value; all metrics are computed on concatenated out‑of‑fold predictions from stratified 5‑fold cross‑validation; 95% confidence intervals were obtained by bootstrap resampling of the OOF set (B = 2000) (AUC via pROC bootstrap). Threshold‑based metrics are from the pooled OOF set at the prespecified probability cut‑off of 0.50.

**Fig. 2:**
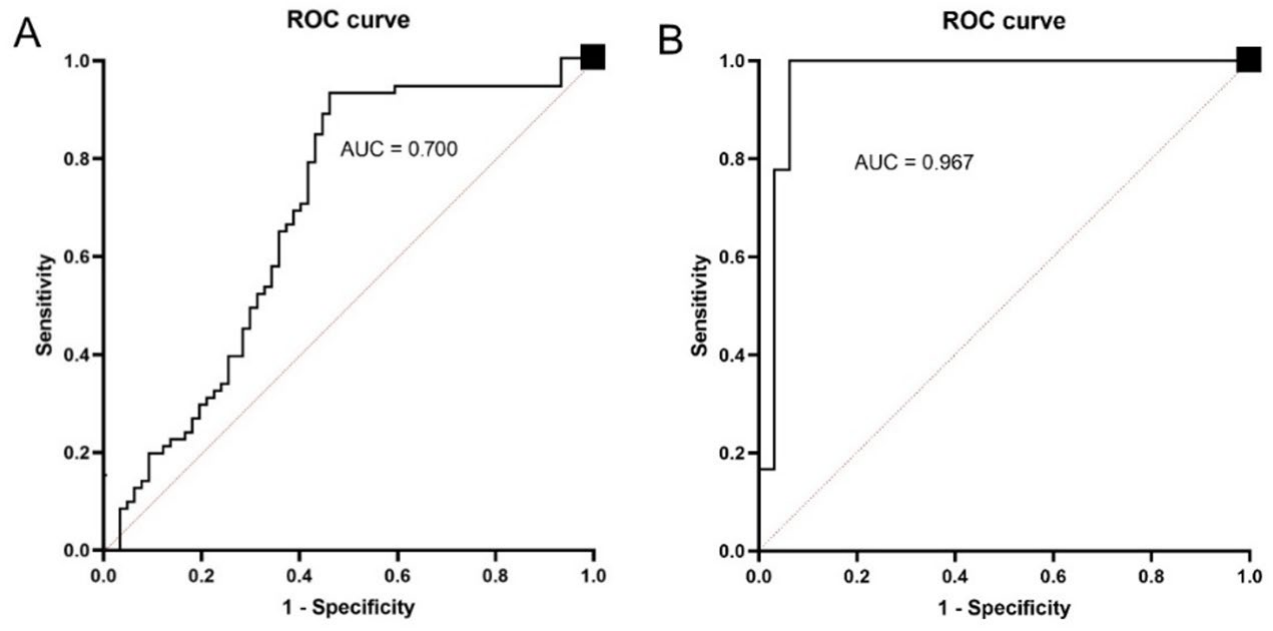
ROC curve derived from out‑of‑fold predictions (**A**) for the logistic regression model, AUC (95% CI) 0.700 (0.565–0.823) and (**B**) ANN model, AUC (95% CI) 0.967 (0.967–1.000), showing true positive rate against the false positive rate (dark line) with the red line representing a random choice.

An AUC (Fig. 2) of 70.0% (95% CI: 56.5;82.3) for LR and 96.7% (95% CI: 96.7;100.0) for ANN, indicates the model’s ability to correctly distinguish a randomly chosen positive case from a randomly chosen negative one.

The mentioned values suggest that the LR model has modest sensitivity and moderate precision, whereas the ANN model is characterised by high discriminant power and generalisation ability. ANN outperforms LR in detecting complications (zero false negatives), making it a better predictive model.

**Fig. 3:**
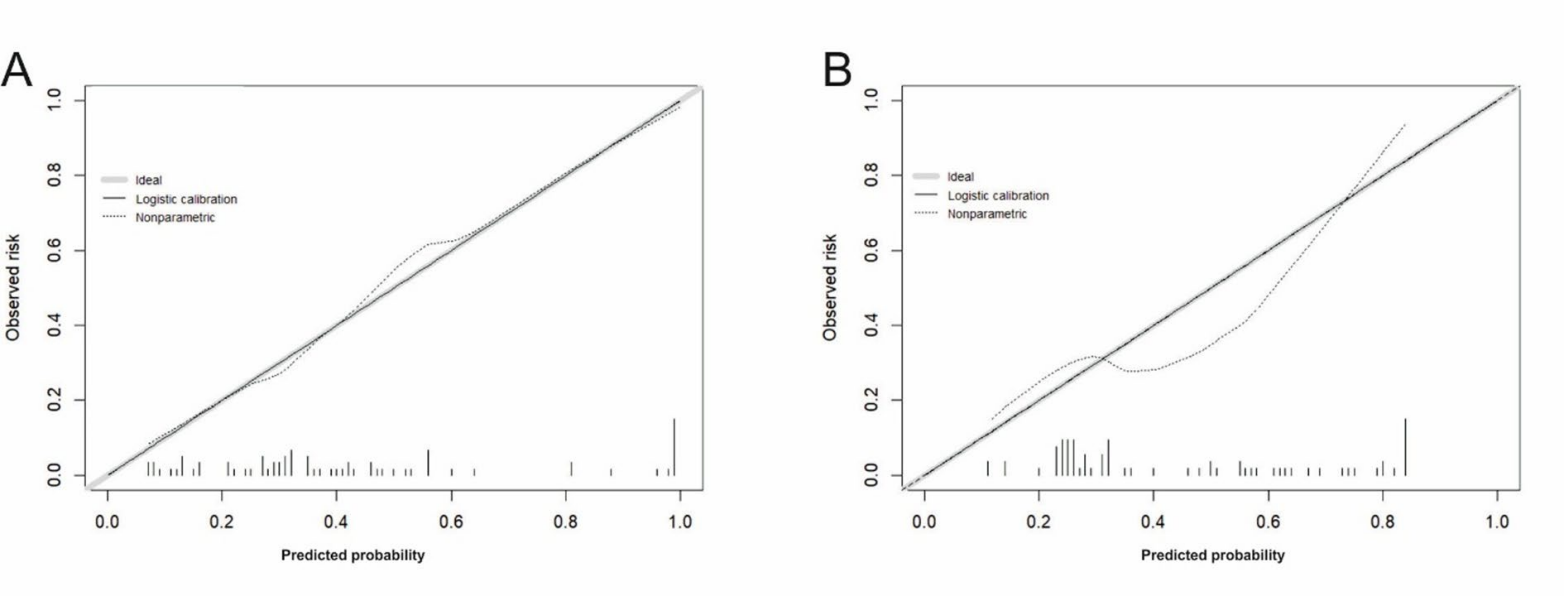
Calibration of predicted probability for (**A**) logistic regression (LR) and (**B**) artificial neural network (ANN). The grey 45° line denotes perfect calibration; the solid line is the logistic recalibration fit (intercept and slope); the dotted line is the non-parametric LOESS curve based on out-of-fold predictions. Rug marks on the x-axis show the distribution of predicted risks (one tick per patient).

The logistic regression model showed good calibration (Fig. 3A; Table 4) with an intercept of -0.052 and slope 0.972, Brier score of 0.189, and ICI (E_avg_) equal to 0.084, while the calibrated ANN (Fig. 3B) achieved acceptable but weaker calibration: intercept − 0.135, slope 0.857, Brier score 0.235, and ICI equal to 0.124. The LOESS curve indicates mild overestimation of risk at mid-range probabilities and underestimation above ~ 0.70. A few observations in the tails (rug marks) make extreme-range deviations unstable.

**Fig. 4:**
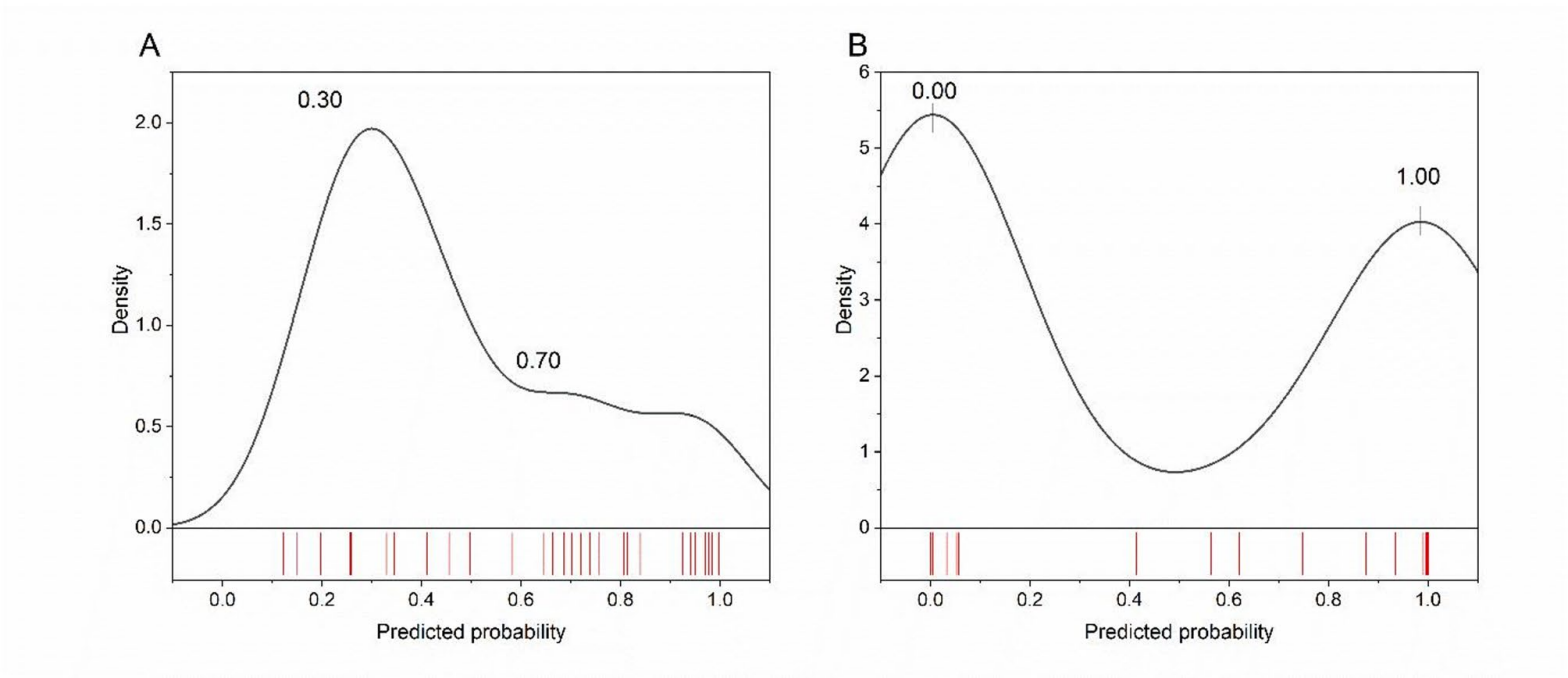
Out‑of‑fold predicted probabilities with kernel density estimates and rug plots for LR (**A**) and ANN (**B**). LR concentrates predictions in the mid‑range; ANN displays more extreme probabilities, indicating greater separation.

Figure 4 displays the out‑of‑fold predicted probability distributions for postoperative complications from (A) logistic regression and (B) the calibrated artificial neural network. Densities and rug marks indicate how predictions are spread across the 0–1 range. The LR model concentrates many cases around intermediate probabilities (0.3–0.7), consistent with greater classification uncertainty. In contrast, the ANN shows a more polarised distribution with mass near the extremes, suggesting clearer separation between cases. These patterns align with the higher discrimination observed in the ANN and explain why LR yields more borderline predictions.

To sum up, both models were developed and assessed under the same internal validation scheme, with evaluation based on pooled out-of-fold predictions. The ANN provided stronger discrimination and case-level detection, yielding fewer borderline probabilities and clearer class separation (Figs. 1, 2 and 4). The LR model offered superior calibration and direct coefficient interpretability, producing probability estimates that align more closely with observed risks (Fig. 3; Table 4). These differences reflect the methods’ assumptions and inputs: LR relates predictors linearly to log odds and was fit on a collinearity-pruned set, whereas the ANN can represent nonlinearities/interactions and was trained on the full candidate set. In practice, ANN is preferable when minimising missed complications is paramount (e.g., perioperative flagging/triage), while LR is preferable when well-calibrated absolute risk estimates and transparency are required (e.g., shared decision making, threshold setting). A pragmatic strategy is to use the ANN for initial screening and the LR estimate to contextualise borderline cases. Prospective external validation is warranted before clinical deployment.

### Characteristics of procedures

The extent of surgical intervention, reflected by the number of procedures performed during CRS, emerged as a significant predictor of postoperative complications in both modelling approaches. While this association was consistent across methods, it warrants further investigation to disentangle the potential interplay between surgical complexity, tumour burden, and perioperative risk.

**Table 5:** Univariate odds ratios (OR) from 2 × 2 tables (exposure: procedure yes/no; outcome: complication yes/no). Holm‑adjusted p‑values; exploratory analysis.

Procedure	OR	*p*-value
Pelvic peritonectomy	1.38	0.704
Diaphragmatic peritonectomy	2.39	0.194
Parietal peritonectomy	1.39	0.669
Liver capsule resection	1.71	0.387
Greater omentum resection	1.99	0.256
Lesser omentum resection	1.91	0.819
Hysterectomy with adnexa	11.67	0.035
Appendectomy	0.72	0.405
Metastasectomy	7.42	0.042
Resection of the rectum/sigmoid colon	2.76	0.304
Splenectomy	3.26	0.124
Right hemicolectomy	2.67	0.512
Cholecystectomy	2.64	0.498
Small intestine resection	1.25	0.937
Pancreas resection	1.01	0.232
Excision of cancer nodules	2.21	0.603

p‑values Holm‑adjusted for 16 comparisons; exploratory analysis; limited power.

To determine which procedure could have the greatest impact on complication occurrence, a univariate 2 × 2 OR analysis (with Holm-adjusted p-values) was conducted for each procedure. The statistics (Table 5) showed that only two procedures significantly increased the risk of complications: hysterectomy with adnexa (OR = 11.67, *p* = 0.035) and metastasectomy (OR = 7.42, *p* = 0.042). Among the 11 patients who underwent hysterectomy with adnexal removal, postoperative complications were observed in 7 cases. These included left ureteral injury, wound infection with purulent discharge, and enteric fistula formation. Similarly, 9 of the 13 patients who underwent metastasectomy experienced postoperative complications, such as intraoperative and postoperative bleeding, surgical site infection, and prolonged postoperative pain.

## Discussion

This study evaluated the ability of two complementary prediction approaches – a LR model and an ANN – to stratify the risk of in-hospital postoperative complications after CRS combined with HIPEC for recurrent OC using pre- and intraoperative information available at the end of surgery. In a single-centre cohort of 71 patients, postoperative complications occurred in nearly half of the cases, underscoring the substantial morbidity associated with this treatment and the need for robust, real-time risk stratification tools.

Consistent with previous reports, CRS with HIPEC in recurrent OC was associated with high rates of postoperative morbidity but no in-hospital mortality in our series^5,6,12–16^. Prior studies have shown that CRS with HIPEC can improve PFS and OS in selected patients, but at the cost of considerable gastrointestinal, infectious and haematological toxicity^3–6,12,15,17,18^. These findings support the view that CRS with HIPEC should be reserved for carefully selected patients and delivered in specialised centres with the infrastructure to manage procedure-related complications.

Traditionally, postoperative risk assessment in this setting has relied on generic indices such as the PCI, CCR, ASA score and comorbidity scales such as the CCI^7,11^. While these measures provide useful baseline information, they were not designed as dynamic, procedure-specific prediction tools for CRS with HIPEC and may fail to capture complex interactions between tumour burden, surgical complexity and physiological reserve^7,11,18–20^. Our analysis supports the hypothesis that combining patient-level factors with detailed intraoperative data can refine risk stratification beyond what is achievable with conventional scores alone^20^.

In the LR model, higher intraoperative blood loss and a greater number of concomitant procedures were independently associated with an increased risk of any postoperative complication, while higher ASA score showed a consistent but less precisely estimated effect. These findings are clinically intuitive and align with previous work suggesting that surgical complexity, extent of peritoneal disease and physiological reserve are key determinants of morbidity after CRS and HIPEC^3–5,7,18,21,22^. From a pragmatic standpoint, the LR model offers transparent, easily interpretable coefficients that can be directly mapped onto modifiable domains such as operative planning, haemostatic strategies and postoperative monitoring intensity.

All performance metrics for both models were derived from stratified 5-fold internal validation, using out-of-fold predictions only, in line with contemporary guidance for prediction model studies^23,24^. Within this framework, the LR model showed moderate discrimination and favourable calibration, with a low Brier score, an intercept close to zero and a calibration slope close to one. This indicates that, on average, LR-based probabilities aligned reasonably well with observed event rates across the spectrum of risk, which is particularly important when the model is used to estimate absolute risk to support shared decision-making or informed consent. Recent perioperative risk studies have similarly emphasised that good calibration is at least as critical as discrimination when prediction models are applied in clinical practice^19,23,25^.

The ANN substantially outperformed LR in terms of discrimination, with a markedly higher AUC and improved classification metrics at the prespecified probability threshold, including very high sensitivity. This suggests that the ANN is particularly suited to a “safety-first” triage role, where failing to identify a high-risk patient is more clinically harmful than unnecessary escalation of care. However, despite Platt scaling within each cross-validation fold, the ANN remained less well calibrated than LR, with a tendency to generate more extreme predicted probabilities. This pattern is consistent with other comparative studies in which ANNs or other machine-learning models achieved superior discrimination but required careful post-hoc calibration to produce reliable absolute risk estimates^19,23,25–29^.

Our findings regarding the superior discriminative performance of ANN compared with LR are in line with prior work in diverse surgical and critical care settings. Other researchers reported that ANNs outperformed LR in predicting postoperative outcomes, including mortality and major complications, by better capturing non-linear interactions between clinical variables^26–28^. At the same time, systematic reviews and comparative studies have shown that in some contexts machine-learning models offer only modest gains over well-specified traditional models, particularly when datasets are small or predictors are limited^30–34^. Taken together, these observations suggest that the relative benefit of ANN over LR is problem-dependent and conditioned by both data complexity and model development procedures.

Future extensions of the logistic regression model could incorporate more flexible specifications, such as restricted cubic splines for continuous predictors, prespecified interactions (e.g., between surgical complexity measures such as number of procedures and blood loss), and penalised logistic regression (ridge or elastic net) fitted within cross-validation. We deliberately did not pursue these extensions in the present small, single-centre cohort to avoid overfitting and to preserve parsimony and interpretability. The observed ANN–LR performance differences should therefore be interpreted conditional on this deliberately simple LR specification.

While explainable‑AI methods such as SHAP can enhance transparency, in this small, single‑centre cohort (*n* = 71; 32 events) with correlated perioperative predictors, we intentionally did not include case‑level post‑hoc explainers in the main text, because their attributions are sensitive to background choices and can be unstable in small samples. Instead, we emphasised methods with stable frequentist properties—out‑of‑fold discrimination and calibration under identical 5‑fold validation for both models, and logistic‑regression coefficients/ORs with 95% CIs—together with within‑fold Platt scaling for the ANN to improve probability calibration. We therefore view SHAP as most informative at external validation; accordingly, we have pre‑specified global and local SHAP analyses for our planned multicentre cohort, where a larger sample size should support reproducible attributions and more actionable clinical narratives.

Both applied methods found that the number of surgical procedures was an important factor in determining complications. Procedure-specific associations were exploratory and not included as predictors due to small-sample EPV constraints and collinearity with surgical-complexity measures; these features are pre-specified for consideration in external multicentre validation.

In practical terms, the complementary profiles observed in our study support a dual-use interpretation. The LR model, with its stable calibration and interpretable predictors, may be preferable when clinicians need to communicate absolute risk or to understand which risk domains drive a patient’s profile. The ANN, conversely, may serve as a high-sensitivity screening tool at the end of surgery to flag patients who warrant ICU admission, extended monitoring or early proactive management of expected complications. Embedding both models in a perioperative pathway – for example, using LR-based risk estimates for preoperative counselling and ANN-based alerts for postoperative triage – could leverage the strengths of each approach while mitigating their respective limitations^19,31,32^.

Beyond model-level performance, our exploratory analysis of individual procedures suggested that certain interventions, particularly hysterectomy with adnexal removal and metastasectomy, were associated with higher complication rates. Procedure-specific associations were exploratory and not included as predictors due to small-sample EPV constraints and collinearity with surgical-complexity measures; these features are pre-specified for consideration in external multicentre validation.

These procedure–outcome associations are also susceptible to confounding by indication. For example, patients selected for metastasectomy typically have a greater tumour burden and may undergo more extensive cytoreduction, so the observed excess risk may partly reflect underlying disease severity rather than the procedure itself. Accordingly, these findings should be regarded as exploratory and not causal. In larger, prospective multicentre cohorts, we plan to use formal causal inference approaches, such as propensity score matching, overlap weighting, and marginal structural models, to better disentangle the effects of tumour burden, surgical complexity, and specific procedures on postoperative morbidity.

In our cohort, hysterectomy with adnexal removal was performed during CRS for ROC in 11 patients (16%), as this procedure had not been carried out during primary surgery—most often for reasons that were undocumented or unclear. The results of this study highlight the importance of including hysterectomy with adnexectomy in the primary surgical management of epithelial OC. Performing this procedure during secondary cytoreduction was associated with a higher rate of postoperative complications, underscoring the potential clinical consequences of omitting it at the initial treatment stage^21,22^. These findings are in line with previous observations linking more extensive pelvic and peritoneal resections with increased operative time, blood loss and postoperative morbidity^3,4,18,22^. Nonetheless, the current study was underpowered to robustly quantify procedure-specific risk, and the multiple comparisons involved increase the likelihood of spurious associations. Accordingly, these signals should be viewed as hypothesis-generating and validated in larger, multicentre series before being translated into procedural thresholds or formal risk scores^20^.

Several limitations merit cautious interpretation of our findings. First, the study was retrospective and conducted in a single high-volume centre, which limits external generalisability. Case-mix, surgical expertise, anaesthetic protocols and postoperative pathways may differ substantially across institutions^5,6,15,16^. Second, the sample size was modest, with a limited number of events relative to the total number of candidate predictors. Although we restricted the LR model to a small set of clinically prespecified variables and used a compact, regularised ANN architecture evaluated via cross-validation, overfitting – particularly for the ANN – cannot be excluded, and the impressive discrimination should be interpreted in the context of wide confidence intervals^19,30,31,33,34^.

Third, the outcome was defined as any in-hospital postoperative complication, including low-grade events. This approach reflects the clinical reality that even minor complications can prolong recovery and consume resources, but it may dilute the model’s ability to specifically distinguish patients at risk of life-threatening events. Future work could explore separate models for any complication and for clinically significant (e.g. Clavien–Dindo grade ≥ III) complications, following approaches used in broader perioperative risk research^19,25^. Fourth, although we carefully avoided post-discharge data leakage by restricting predictors to pre- and intraoperative variables available at the end of surgery, we did not include longer-term outcomes such as 90-day morbidity, readmissions or oncologic endpoints, which would require a different design and follow-up strategy.

Finally, this study reports only internal validation. External validation in independent cohorts is essential before any deployment in routine care^19,24,30,35^. We have therefore pre-planned a multicentre validation study to evaluate transportability, re-calibrate the models if necessary and, in a larger sample, explore more advanced model-agnostic explanation techniques such as SHAP in a statistically robust manner^36–39^. Until such validation is complete, both models should be considered decision-support research tools rather than ready-to-use clinical calculators.

## Conclusions

Our results suggest that combining traditional statistical modelling with modern machine learning can meaningfully improve perioperative risk stratification after CRS with HIPEC for recurrent OC. A well-calibrated, interpretable LR model and a highly discriminative ANN appear to offer complementary advantages that, if confirmed externally, could support more tailored allocation of intensive care resources, earlier recognition of high-risk patients and ultimately safer delivery of this demanding but potentially beneficial treatment.

## Data Availability

De‑identified data are available upon reasonable request, subject to institutional approvals.

## Code availability

Analysis code (R scripts and ANN notebooks) is available upon reasonable request from the corresponding author.
